# Development and maintenance of consensus recommendations on pediatric outpatient antibiotic therapy in Germany: a framework for rational use

**DOI:** 10.1007/s00431-024-05964-y

**Published:** 2025-01-23

**Authors:** Janina Soler Wenglein, Arne Simon, Reinhard Berner, Holger Brockmeyer, Johannes Forster, Eckard Hamelmann, Wolfgang Klein, Johannes Liese, Jennifer Neubert, Johannes Pfeil, Hanna Renk, Tobias Tenenbaum, Nicole Toepfner, further ABSaP Working Group members, Jakob Armann, Chris Boethig, Oezguer Dogan, Johannes Huebner, Jost Lange, Thomas Lenz, Steffen Lueder, Gerhard Moeller, Thomas Parlowsky, Julia Prusseit, Stefan Reinke, Tilmann Schober, Stefan Trapp, Ulrich von Both, Anne-Sophie Yussif, Markus Hufnagel, Roland Tillmann

**Affiliations:** 1 Department of Pediatrics, Protestant Hospital of the Bethel Foundation, Medical School and University Medical Center OWL, Bielefeld University, Bielefeld, Germany; 2 Laboratory of Experimental Pediatric Pneumology and Allergology, Center for Biomedical Education and Science (ZBAF), Department of Human Medicine, Faculty of Medicine, Witten/Herdecke University, Witten, Germany; 3 Medical School OWL, Bielefeld University, Bielefeld, Germany; 4 Pediatric Oncology and Hematology, Pediatric Infectious Diseases, Children’s Hospital, Saarland University Medical Center, Homburg, Germany; 5 Department of Pediatrics, Faculty of Medicine and University Hospital Carl Gustav Carus, Technische Universität Dresden, Dresden, Germany; 6 Praxis für Kinder und Jugendliche Holger Brockmeyer, Hamburg, Germany; 7 Institute for Hygiene and Microbiology, University of Würzburg, Würzburg, Germany; 8 Praxis für Kinder- und Jugendmedizin Wolfgang Klein, Augsburg, Germany; 9 Department of Pediatrics, University Hospital Würzburg, Würzburg, Germany; 10 Praxis für Kinder- und Jugendmedizin Jennifer Neubert, Neuss, Germany; 11 Department of Pediatric Oncology, Hematology and Clinical Immunology, Center for Child and Adolescent Health, Medical Faculty, Heinrich Heine University, Düsseldorf, Germany; 12 Kinder- und Hausarztpraxis Johannes Pfeil, Schwaigern, Germany; 13 Department of Pediatric Neurology and Developmental Medicine, University Children’s Hospital, Tübingen, Germany; 14 Child and Adolescent Medicine, Sana Children’s Hospital Lichtenberg, Academic Teaching Hospital Charité-Universitätsmedizin Berlin, Berlin, Germany; 15 Division of Pediatric Infectious Diseases and Rheumatology, Department of Pediatrics and Adolescent Medicine, Medical Faculty, University Medical Centre, University of Freiburg, Freiburg, Germany; 16 Praxis für Kinder- und Jugendmedizin Roland Tillmann, Ärztenetz Bielefeld, Bielefeld, Germany

**Keywords:** Antibiotic prescription, Antibiotic stewardship, Outpatient pediatrics, Standard of care, Best practices

## Abstract

In pediatric outpatient care, overuse and misuse of antibiotics is linked to a high risk of adverse events and increased antibiotic resistance. In 2019, building upon the work of the Antibiotic Therapy in Bielefeld (AnTiB) project (founded in 2016), a collaboration among the AnTiB, the German Society for Pediatric Infectious Diseases (DGPI), and the Professional Association of Outpatient Pediatricians and Adolescent Physicians (BVKJ) was established to develop consensus recommendations for antibiotic therapy in pediatric outpatient settings in Germany. This working group became the Antibiotic Stewardship in Outpatient Pediatrics (ABSaP). ABSaP institutes recommendations for antibiotic use in outpatient pediatric settings in Germany and updates them regularly. The ABSaP guidelines recommend restrictive, targeted, evidence-based prescription practices for antibiotics used to treat common infections among pediatric outpatients. This expert-driven, pragmatic, best-practice guidance is designed to be a living document. ABSaP’s most recent update was published in March 2024. The recommendations aim to guide and standardize rational antibiotics use by emphasizing the importance of avoiding unnecessary prescriptions, while also promoting the application of narrow-spectrum antibiotics, short-duration therapy, and watchful waiting, when treating mild, self-limiting infections in children without significant risk factors.

*Conclusions*: ABSaP’s guidelines may provide a model for others, as well as offer a basis for discussing practical, effective antibiotic stewardship (ABS) measures in pediatric primary care. An international, expert consensus on ABS for pediatric outpatients could help promote a culture of responsible antibiotic use, improve prescribing safety, and contribute to broader ABS efforts.

**Table:** 

**What is Known:** • *Overuse and misuse of antibiotics are associated with adverse events and increased antibiotic resistance, prompting the need for effective antibiotic stewardship initiatives.* • *The Antibiotic Stewardship in Outpatient Pediatrics (ABSaP) working group was established to develop and regularly update evidence-based best practice recommendations for the targeted and restrictive use of antibiotics in pediatric outpatient settings in Germany.*	
**What is New:** • *The most recent update of the ABSaP guidelines was published in March 2024, emphasizing the importance of narrow-spectrum antibiotics, short-duration therapy, and watchful waiting for mild infections, aiming to standardize rational antibiotic use in children.* • *The ABSaP guidelines have become widely accepted in Germany and are now being translated into English to foster international dialogue and collaboration on antibiotic stewardship in pediatric primary care, potentially serving as a model for similar initiatives in other countries.*

**Supplementary Information:**

The online version contains supplementary material available at 10.1007/s00431-024-05964-y.

## Introduction

Following a variety of antibiotic stewardship (ABS) initiatives instituted in Germany over the last 15 years, antibiotic prescription rates for children have been steadily declining [1–3]. In the past, antibiotic use in German pediatric outpatient settings was high in comparison to that of other European countries [2]. For Germany, however, the period 2010–2018 showed a marked reduction [1]. Even though age-specific survey data comparing antibiotic prescribing practices in outpatient pediatric settings is not collected on a Europe-wide basis, the European Surveillance of the Antimicrobial Consumption Network (ESAC-Net) [4] does make available general population data regarding outpatient antibiotic use in Europe [5].

Despite the well-documented, positive impact of ABS on the pediatric population [6–8], the individual and regional variations in both antibiotic prescription rates and choice of antibiotics prescribed for infections in otherwise healthy children without pre-existing chronic conditions, comorbidities, or other risk factors remain pronounced [9–11]. For example, in a cross-border comparison of outpatient pediatric antibiotic use published in 2016, antibiotic prescription rates were shown to be higher in Germany than in the Netherlands [12]. In a 2024 study examining Danish and German birth cohorts, Germany was shown to have lower antibiotic prescription rates, but a less favorable choice of antibiotics with respect to treatment spectrum and potential side effects [13]. Therapeutic approaches vary considerably with respect to overall antibiotic prescribing practices [12], use of broad- vs. narrow-spectrum antibiotics [14], choice of point-of-care diagnostic tools [15–18], watchful waiting strategies [19], and how diagnostic and prognostic uncertainties become dealt with [20].

Uncertainties are part of everyday practice, particularly in primary care. The fact that no diagnostic test can reliably distinguish between viral and bacterial infections (e.g., in cases of tonsillitis, otitis, airway infections) means that diagnostic uncertainty is often unavoidable. Prognostic uncertainty entails the risk on an individual level of serious complications developing during an infection (e.g., meningitis, sepsis with organ failure, mastoiditis, complicated pneumonia). How individual physicians cope with such uncertainties, as well as whether parents express the desire to receive antibiotic treatment for their child’s illness, often affects decisions in favor or against antibiotic treatment [21–23].

Many physicians do not perceive the emergence of antibiotic-resistant pathogens as relevant to their own personal medical practices [24–26]. When it comes to inappropriate antibiotic prescribing and related antibiotic resistance patterns, pediatricians — even those with very high antibiotic prescription rates — tend to pass the blame to general practitioners (GPs), ENT-specialists, urologists and/or inadequately trained or understaffed pediatric emergency service in their regions [27]. This notwithstanding, the confirmed correlation between broad use of antibiotics and prevalence of multiple antibiotic-resistant infectious organisms (MRO) [28–34] makes it clear that the impact of personal prescription practices must be considered on a global health scale. For example, MRO development includes macrolide-resistant pneumococci, group A streptococcus (GAS) [35, 36], penicillin- and amoxicillin-resistant pneumococci [37], and amoxicillin- and macrolide-resistant *Haemophilus influenzae* [38]. Furthermore, inappropriate use of antibiotics may expose patients to undesirable risks and side effects, including abdominal pain, nausea, vomiting, antibiotic-associated diarrhea [39], drug exanthema, and autoimmunological reactions [40, 41]. More severe complications related to antibiotic misuse or overuse — including enterocolitis caused by *Clostridioides difficile* [42, 43], acute liver failure [44], and severe skin reactions [45] — are rare but nevertheless important to keep in mind.

Because rational use of antibiotics is a significant challenge in everyday outpatient pediatric care, the ABSaP Working Group on Antibiotic Stewardship in Outpatient Pediatrics (AG ABSaP) launched a project to formulate expert-driven, evidence-based, best practice recommendations for the treatment of standard outpatient infections — guidelines serving to promote rational antibiotic prescribing, while also addressing the basic requirements of primary pediatric care in Germany. With the current report, we hope to stimulate international dialogue and exchange on antibiotic stewardship in primary pediatric care. National guidelines in Europe mainly are available in local languages, which can create barriers to broader collaboration and knowledge sharing. By making our guidelines accessible to an international audience, we hope to contribute to a public discourse that fosters collaboration and best of care in the field of pediatric primary care. We are eager to share our experiences and insights, as we believe that such exchanges can enhance the quality of care provided to children, ultimately benefiting public health on a larger scale.

However, successful antibiotic stewardship is not merely about establishing and disseminating expert information and guidelines. For the successful implementation of ABS guidelines, a strategy customized to the regional culture of antibiotic prescribing habits is needed. The approach taken also needs to factor in the fact that patients are often treated by different care providers across multiple health sectors.

The AnTiB (Antibiotic Therapy in Bielefeld) project [46], initiated in 2016 by a local network of physicians providing pediatric care, launched a local, multidisciplinary discussion platform to discuss different standards and habits operating in different medical specialties (e.g., GPs vs. pediatricians vs. ENT physicians) [47]. The outpatient medical sector in Germany is focused on specialized care and principally organized according to specialty. Although children and adolescents primarily receive outpatient treatment from pediatricians, others such as general practitioners, ENT physicians, and ophthalmologists, among other medical specialists, also can be involved in pediatric care [48]. Outside standard office hours, primary medical care in Germany often is provided by hospital emergency departments. Therefore, when developing treatment guidelines, the voices of a wide range of local players were called upon to provide multidisciplinary expert advice. This collaborative, interdisciplinary approach to developing guidelines is particularly beneficial in that it can help reduce frequency of contradictory expert recommendations, while also leading to higher patient and parent satisfaction. For these reasons, ABSaP has worked to promote a culture of responsible antibiotic use that extends beyond primary care pediatricians to include all physicians and health care workers involved in treating infections among children and adolescents at secondary and tertiary care facilities [49].

In recent years, as the ABSaP guidelines increasingly have become implemented in Germany, the recommendations have become broadly accepted. With the current report, we hope to stimulate international discussion about ABS for pediatric outpatients, as well as to potentially pave the way for future international outpatient ABS guidelines and programs combating antibiotic resistance on a broader scale. With this, we support the WHO strategy regarding infection prevention and control, as well as the global action plan on antimicrobial resistance [50, 51].

## Methods and content considerations

This report and its supplement describe the recommendations established by the ABSaP Working Group. Developed with input from the Working Group Antibiotic Therapy in Bielefeld (AnTiB), the Professional Association of Outpatient Pediatricians and Adolescent Physicians (BVKJ), and the German Society for Pediatric Infectious Diseases (DGPI), the guidelines are the result of an intensive, ongoing consensus process that began with AnTiB in 2016 and since 2019 have involved all ABSaP Working Group members. This process involves collaboration between outpatient pediatricians, microbiologists, ABS experts, and experts on infectious diseases. The goal is to continuously reevaluate [52–54], re-consent, and update best practice guidelines for treating pediatric outpatients with common infections in German primary care settings. The recommendations are jointly developed and agreed upon based on literature research and the collection of information regarding the national antibiotic resistance situation, as well as on practical considerations and feedback from primary care pediatricians. Members of the Working Group prepare concise recommendations based on the current international literature. In a series of online meetings, each proposal is jointly discussed in order to agree upon a consensus statement. End users are actively encouraged to provide comments and questions to the ABSaP Working Group. For example, one recent update based upon newly published evidence was the recommendation for a shorter duration of antibiotic therapy when treating pneumonia [55].

ABSaP recommendations are published on the DGPI homepage, regularly presented at conferences and workshops, as well as at lectures. Members of the ABSaP Working Group frequently collaborate with regional stakeholders in discussion rounds where print versions of the guidelines are provided. In addition, the recommendations have been disseminated via online training courses and in German medical journals. Finally, the DGPI offers bi-monthly digital training courses on pediatric infectious disease topics through which updates on antibiotic therapy regularly are reported.

Because the ABSaP recommendations are oriented toward outpatient therapy, they have limited applicability in the treatment of more complex conditions. Specifically, the target patients are children ≥ 1 month old who have no pre-existing conditions or other risk factors, and who are not experiencing severe courses of disease.

One main objective of the ABSaP recommendations is to avoid the initiation of unnecessary antibiotic therapy and/or to immediately stop unnecessary antibiotic therapy in circumstances where a bacterial infection reliably has been ruled out. In cases where an immunocompetent patient has mild, self-limiting bacterial diseases, the guidelines suggest that antibiotic therapy be avoided. If antibiotic treatment against infection is indicated, certain aspects should be considered: 1) Antibiotic therapy should be prescribed as short a time as possible. 2) The antibiotic therapy spectrum should be as narrow as possible. 3) Use of critical antibiotics (defined in relation to risk of developing resistance and/or side effects) should be avoided. 4) If allergies to antibiotics hinder the use of small-spectrum antibiotics, the presence of those allergies should be diagnostically verified or ruled out before proceeding. 5) The antibiotic prescription should be of good quality. This requires accurate information on dose, duration of therapy, and recommended intake. 6) Topical antibiotic applications should be avoided due to their potential to induce resistance. Instead, local antiseptics are preferred. 7) Information regarding anticipated course of disease, red flags, and rules for readmission to a medical facility should be provided to patient and their parents. 8) If reasonable and possible, delayed prescriptions and/or watchful waiting should be considered options.

Important to keep in mind is the fact that antibiotic prescriptions take place within a social medical framework. This has implications for both medical practice and public health. For these reasons, the ABSaP recommendations can — and indeed should — be adapted to local and regional circumstances, because local resistance situations may require alternative antibiotics to be chosen. This especially applies when adapting the ABSaP recommendations to countries where the resistance situation may be different from that in Germany.

The ABSaP recommendations are grouped by the type of disease and provide short bullet-point information regarding diagnostic and therapeutic procedures. The recommendations are intentionally concise for practical purposes, while also explicitly acknowledging that they do not cover every nuance of pediatric care. We have included hints on areas that may require further exploration, such as the term “strict indication.” The four-page, pocket format is concise, regularly updated, and freely available for download via the homepage of the DGPI [54]. Recommendations for each antibiotic therapy are presented according to the scheme shown below. Where applicable, the preferred therapy is highlighted in grey:
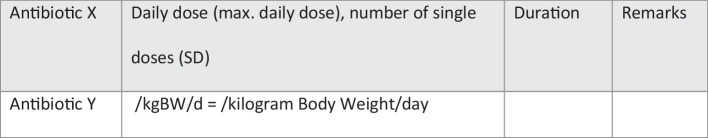


### Ethical approvement

No ethical approval is required for this work. The recommendations are a result of an intensive, consensus process, including a peer-review, and are aimed at improving the quality of medical care and patient safety. These recommendations are in line with the principles of the Declaration of Helsinki.

### ABSaP guidance document

The document itself (see Supplement) is divided into six main sections: (1) respiratory tract infections, (2) urinary tract infections, (3) skin infections, (4) ophthalmic infections, (5) intestinal infections, and 6) surgical infections in pediatric primary care.

#### ENT and respiratory tract infections

ENT and upper respiratory tract infections in children < 6 years old are one of the most common reasons for visits to pediatricians, emergency care units, and general practitioners [56, 57]. The majority of infections observed in this context (i.e., acute otitis media, rhinitis, sinusitis, tonsillopharyngitis, subglottic laryngitis, acute bronchitis) are viral infections. Only a much smaller proportion are bacterial infections, ones which frequently result from a previous viral infection [58–62]. The ABSaP guidelines contain recommendations for treatment of tonsillopharyngitis, recurrent GAS tonsillopharyngitis, acute otitis media, perforated otitis media, otorrhea with tympanostomy tube, otitis externa, acute sinusitis, acute bacterial lymphadenitis colli, pseudocroup, laryngitis, acute (obstructive) bronchitis, community-acquired pneumonia, and whooping cough (pertussis). RSV bronchiolitis and influenza infections are highlighted as important differential diagnoses. These and other viral infections identified should only be treated symptomatically. Information regarding potentially preventable complications also is provided. In addition, recommendations for standard diagnostic procedures are included. For example, in cases of tonsillopharyngitis diagnosed using the modified McIsaac Score [63], the guidelines suggest that a rapid antigen test for GAS be considered. Antibiotic therapy in tonsillopharyngitis is only indicated in very specific circumstances: patient age of ≥ (2–)3 years, severe illness, fever, painful cervical lymphadenopathy, and absence of signs for a viral infection (e.g., cough, conjunctivitis). Considerations for primary prevention measures are referenced separately (e.g., vaccine preventability in pertussis).

#### Urinary tract infections (UTI)

By the age of 6, over 7% of all girls and 1.6% of boys have experienced at least one UTI episode [64, 65]. Cystitis can manifest with dysuria, pain in the lower abdomen, and other urinary abnormalities. Fever and flank pain indicate an ascending urinary tract infection [66]. Among infants ≤ 2 years old who visit emergency medical departments and present with fever of unknown origin, approximately 7% have been diagnosed with a UTI [67]. For diagnostic purposes, clean collection of urine specimens is essential [68, 69].

With a reliable diagnosis, short-duration antibiotic treatment of uncomplicated UTIs in children ≥ 2 years of age is safe and efficient [70]. In the ABSaP guidelines, the UTI section addresses uncomplicated cystitis, pyelonephritis, and prophylaxis for urinary tract infections [69, 71–73]. When considering treatment for uncomplicated cystitis with unclear diagnosis (e.g., differential diagnosis vulvitis), as well as in cases where patients have mild symptoms and no fever, no antibiotic therapy is recommended. This recommendation is based upon data from adult female patients [74]. Information regarding potentially preventable complications also is provided. In addition, recommendations for diagnostic procedures (e.g., urine culture) are included, along with their potential impact on de-escalation of antibiotic therapy in accordance with the antibiogram. For UTI prophylaxis, the guidelines emphasize strict indication and avoidance of cephalosporins (when possible). The strict indication for prophylaxis refers to carefully considering the individual risk of pyelonephritis recurrence and risk of pyelonephritic renal parenchymal damage, depending upon age, higher grade of vesicoureteral reflux, and history of previous UTI, among other factors.

#### Skin infections

The skin infections section of the ABSaP guidelines covers superinfected atopic eczema, impetigo contagiosa, insect bites, perianal GAS dermatitis, and cutaneous Lyme disease. Recommendations for antiseptic [75–77] and anti-inflammatory [78, 79] local therapy are provided. The guidelines highlight the importance of using antiseptic ointments instead of topical antibiotics in mild skin infections. Since *Staphylococcus aureus* is the most common pathogen [80] in impetigo contagiosa, first generation cephalosporins are recommended as first choice antibiotic therapy, when necessary [81, 82]. In perianal streptococcal dermatitis, first-generation cephalosporins or penicillin is treatment options, with follow-up visits recommended due to the high risk of relapse [83, 84]. Children ≥ 8 years old who present with erythema migrans should be treated with doxycycline as a first choice [85]. Suggestions to add the antibiotic therapy of erysipelas and cellulitis are well received and will be added to the next update.

#### Ophthalmic infections

The ophthalmic infections section addresses lacrimal duct stenosis, conjunctivitis, and hordeolum.

Among newborns, a diagnosis of lacrimal duct stenosis is common, but usually this is a self-limiting disease [86–88]. Purulent conjunctivitis among children often is caused by bacterial pathogens, but also is usually self-limiting, besides which antibiotic eye drops do not offer significant benefits [89, 90]. The same applies to hordeola [91]. The guidelines stress the importance of strict indication when prescribing antibiotic eye drops due to their indirect effect on antibiotic resistance of the ophthalmic and nasopharyngeal flora [92, 93].

#### Intestinal infections

The intestinal infections section addresses (hemorrhagic) gastroenteritis. Here, the indications for antibiotic therapy only rarely exist; generally speaking, antibiotic therapy usually is not needed in these cases [94].

#### Surgical infections

The surgical infections section covers a collection of typical (surgical) diagnoses that regularly occur in pediatric outpatient practice: panaritium, abscess, superficial wounds, minor wound infections, and balanitis. Because evidence for optimal therapy for these infections is lacking, the recommendations provided are based upon clinical experience and in alignment with published clinical recommendations [95, 96]. Primarily, surgical and antiseptic therapy is recommended, along with follow-up observation. Additional information on bite wounds is included, because these require special attention with respect to vaccination. For deep wounds, facial bites, or wounds above a tendon or bone, antibiotic prophylaxis should be considered [97].

## Discussion

Providing evidence-based, expert guidance in the diagnosis and treatment of common infections among pediatric outpatients is a core element of antimicrobial stewardship [98–103]. Even though guidelines for managing pediatric infectious diseases are known to be available in nearly all European countries [104], a PubMed search returns only a handful of English-language articles concerning pediatric outpatient therapy guidelines for common outpatient infectious diseases in Europe. A majority of the guidelines issued have been published elsewhere in country-specific national languages. The ABSaP Working Group wanted to open our Germany-specific discussions to a broader audience and thereby learn from the strategies and experiences of other pediatric ABS groups worldwide. For this reason, we decided to translate the German ABSaP guidance into English and make it publicly available [105, 106].

The ABSaP guidance differs from other national ABS guidelines in several important respects. Regarding the choice of antibiotics against GAS tonsillopharyngitis, both the German and Spanish consensus guidelines [107] clearly recommend penicillin. However, in Switzerland, amoxicillin is recommended due to the limited availability of penicillin [108]. For afebrile cystitis, Swiss recommendations also include trimethoprim-sulfamethoxazole or amoxicillin-clavulanic acid [109]. Amoxicillin resistance rates for *Escherichia coli* isolates are high in Germany, as well as across Europe [110]. But based on ABS considerations, the ABSaP guidelines refrain from recommending the use of a broad-spectrum product such as amoxicillin-clavulanic acid and instead recommend trimethoprim (first choice) or nitrofurantoin (second choice) for cystitis. In addition, the ABSaP recommendations explicitly spare antibiotic ear drops, topical skin antibiotics, and antibiotic eye drops. This is in contrast to the NICE guidelines for otitis media [111] and impetigo contagiosa [112], as well as to the National Health Service Greater Glasgow and Clyde guidelines for conjunctivitis [113]. While Swiss recommendations do not suggest an age restriction for doxycycline in the treatment of erythema migrans, the ABSaP recommendations maintain an age restriction for doxycycline for children < 8 years old, this despite recent evidence from the U.S. suggesting its use may be safe in young children [114].

Local and temporal variations in resistance patterns, along with international differences, require ABSaP guidance to be regularly updated. Furthermore, adjustments to dosages may be necessary, depending upon local resistance situations. When prescribing amoxicillin, for example, pneumococci with reduced penicillin susceptibility [115] may require dosage adjustments [116, 117]. In addition, the introduction of new conjugate vaccines covering a wider range of pneumococcus serotypes [118, 119], along with vaccines against respiratory syncytial virus (RSV), may have an important impact on the prevalence of pediatric respiratory tract infections. In Germany, the introduction of PCV7 and PCV10 nearly eliminated the contribution of macrolide-resistant pneumococci to invasive pneumococcal infections [120]. The effect of PCV15 and PCV20 on resistance patterns must be observed carefully, as this too may lead to the need to adapt ABSaP recommendations.

Also changing the equation for pediatric ABS guidelines are differences in how patients may access healthcare locally. In countries where pediatric patients visit general practitioners rather than going to outpatient pediatricians, as is the case in Germany, ABS guidelines may need to be adapted so that they are less pediatric-specific ABS guidelines — this despite the fact that pathogen spectrums vary significantly with patient age. Another important aspect is antibiotic availability. When the threshold for access to antibiotics is low, and/or when narrow-spectrum beta-lactam antibiotics are unavailable, use of broader spectrum beta-lactam antibiotics may increase [121].

In order for antimicrobial therapy to be improved, it is urgent that both public health associations and health care organizations be pushed to focus more keenly on antimicrobial stewardship programs in outpatient pediatric settings. The Centers for Disease Control and Prevention (CDC) recently has emphasized the issue by providing detailed guidance for the development and implementation of outpatient ABS programs [54], efforts which by definition are complex and multifaceted. One aspect the CDC highlights is the importance of the early involvement of primary care providers in assuring the practicability and acceptance of ABS by using “bottom-up” rather than “top-down” approaches. Not only should ABS guidelines be broadly disseminated; they also should be actively implemented in daily practice at the local level. The significant local variability of antibiotic prescriptions strongly suggests the influence of what has been termed “local prescribing cultures” [122]. For this reason, it is particularly important that locally adapted guidelines become implemented — ones that concretely demonstrate the benefits of ABS and improved prescription safety — because these then can serve as tools for achieving compliance and acceptance.

Data from the AnTiB project showed that in Germany, antibiotic prescription rates steadily decreased during the period 2015–2018, a response that followed the active dissemination of AnTiB/ABSaP guidance [3, 11] and led to a further standardization of prescribing behaviors, including in relation to the increased use of narrow-spectrum antibiotics [11].

To date, all evidence points towards the ABSaP guidelines having had a distinctly positive effect on outpatient antibiotic prescribing behaviors in Germany [11]. Due to the impacts of the COVID-19 pandemic, however, long-term observation of these trends remains needed in order to eliminate the possibility of distortion. In order to react to evidence of new microbial resistance patterns with the greatest degree of flexibility, as well as to be able to respond to changes in prescribing behaviors and patient outcomes, it is critical that ABSaP guidelines be understood and managed as living recommendations. Additionally stimulated by international discussions on pediatric outpatient ABS recommendations, the ABSaP Working Group aims to further improve, adapt, and share the best ABS practices available.

## Supplementary Information

Below is the link to the electronic supplementary material.

Supplementary file1 (PDF 322 KB)

## Data Availability

No datasets were generated or analyzed during the current study.

## Abbreviations

ABS: Antibiotic stewardship
ABSaP: Antibiotic Stewardship in Outpatient Pediatrics (Working Group)
AnTiB: Antibiotic Therapy in Bielefeld
BVKJ: Professional Association of Outpatient Pediatricians and Adolescent Physicians
CDC: Centers for Disease Control and Prevention
d: Day
DGPI: German Society for Pediatric Infectious Diseases
dr: Drops
ESAC-Net: European Surveillance of the Antimicrobial Consumption Network
GAS: Group A streptococcus
GP: General practitioner
kgBW: Kilogram body weight
Mo: Months
MRO: Multiple antibiotic-resistant infectious organisms
PCV: Pneumococcal conjugate vaccine
RSV: Respiratory syncytial virus
SD: Single dose
UTI: Urinary tract infection
We: Weeks
