# Supplementary material for: Development and maintenance of consensus recommendations on pediatric outpatient antibiotic therapy in Germany: a framework for rational use
Source: Eur J Pediatr. 2025 Jan 23;184(2):149. doi: 10.1007/s00431-024-05964-y (PMC11754357; doi:10.1007/s00431-024-05964-y)
Supplement: Supplementary file 1 — Supplementary file1 (PDF 322 KB) [file 431_2024_5964_MOESM1_ESM.pdf]

Recommendations for antibiotic therapy in outpatient pediatrics 2024

The following recommendations, based on current guidelines, provide a **concise standard of care** for common infectious diseases in pediatric outpatient settings. These recommendations are not intended to replace the physician's evaluation and decision-making in individual patient cases. Treatment strategies that differ from the recommendations below may be necessary in specific situations but should still be carefully considered and justified by clinicians. If deviations from the recommended treatment become more frequent, this is a strong indication that the recommendations should be reviewed and treatment strategies realigned so as to be in closer accordance with them.
When complex conditions are present — for example **underlying diseases, complicated courses of disease, early infancy, previous antibiotic treatment, travel abroad, etc. —** the recommendations are of limited applicability. No guarantee of successful outcome can be given for the contents of these recommendations, particularly with regard to dosage. Feedback to the editors is welcome! (email: abs-ambulante-paediatrie@dgpi.de)

Main goals of the current recommendations

Reduction of antibiotic (AB) prescriptions:

- Avoid unnecessary AB therapy and/or immediately stop AB if unnecessary.
- Keep AB therapy as short-term as possible and as narrow-spectrum as possible.
- Avoid AB therapy for mild, self-limiting bacterial diseases in immunocompetent patients.
- "Watchful waiting": In unclear situations without any special risk constellation, schedule short-term follow-up visit.
- Consider "delayed prescription": e.g., for acute otitis media.
- Reduce topical AB therapy, e.g., for skin and eye infections.

Improve the quality of AB prescriptions:

- Specify exact dosage, duration of therapy and conditions of use (in relation to meals!) as part of the prescription instructions.
- Avoid prescription of critical AB, only using them selectively, e.g.:
  - Cephalosporins: especially cefuroxime p.o. due to poor oral bioavailability and risk of MRGN development.
  - Macrolides: especially azithromycin due to long half-life and development of resistance.
- If allergy to antibiotics is suspected, seek clarification and/or make referral to an allergy specialist (“de-labeling”).

Presentation of AB therapy according to the following scheme (where applicable the preferred therapy is highlighted in gray):

**Antibiotic X**	**Daily dose (max. daily dose), number of single doses (SD)**	**Duration**	**Remarks**
Antibiotic Y	/kgBW/d = /kilogramm Body Weight/day		

1. Respiratory tract infections

1.1 Tonsillopharyngitis

- No AB therapy, no throat swab recommended if: age <(2-)3 years, mild illness, evidence of viral infection (cough, rhinitis, conjunctivitis, hoarseness, stomatitis, signs of EBV infection, etc.); follow-up if necessary.
- AB therapy is *not* justified: to avoid purulent or immunological secondary diseases, to avoid infections in contact persons (prophylactic antibiotics), to detect *Haemophilus influenzae* or *Staphylococcus* aureus in a swab, high ASL titer.
- Consider Strep A rapid test and AB therapy for: age ≥(2-)3 years, severe illness, fever, painful cervical lymphadenopathy, no cough, no conjunctivitis (reflecting high probability of GAS tonsillopharyngitis according to McIsaac score)

**Penicillin V**	50.000-100.000 IU/kgBW/d (max. 3 Mio. IU) in 2-3 SD	5 – 7 d	not with meals
Penicillin V	100.000 IU/kgBW/d (max. 3 Mio. IU) in 2-3 SD	10 d	for **recurrence**
Benzathin-Penicillin	50.000 IU/kgBW/d (max. 1,5 Mio. IU) in 2 SD	5 – 7 d	
Clarithromycin	15 mg/kgBW/d (max. 1 g) in 2 SD	5 – 7 d	If allergic to Penicillin

After GAS infection: No routine check of ECG and urine status is recommended.
Determination of ASL titer only in case of suspected immunological secondary diseases such as acute rheumatic fever or post-streptococcal glomerulonephritis.

1.1.1 Recurrent GAS tonsillopharyngitis

- Strict indication, consider differential diagnoses: viral infections, periodic fever syndromes, etc.

Clindamycin	30 mg/kgBW/d (max. 1,8 g) in 3 SD	10 d	

1.2 Acute otitis media (AOM)

- **Symptomatic** therapy (NSAR, decongestant nasal drops) is usually sufficient if **short-term follow-up** is possible.
- Offer AB Therapy in infants < 6 months, severe AOM, protracted AOM (> 48-72 hours duration) and underlying disease.

Amoxicillin	50 mg/kgBW/d<br>(max. 3 g) in 2-3 SD	7 d (< 2 years)<br>5 d (≥ 2 years)	Preferably in pharmaceutical suspension,<br>drink plenty of fluids

1.2.1 Perforated otitis media

- If the patient is in *good clinical condition:* no initial AB therapy required, follow-up after 2-3 days.
- Offer antibiotics in case of fever, severe otalgia, persistent otorrhea:

Amoxicillin	50 mg/kgBW/d (max. 3 g) in 2-3 SD	7 d (< 2 years) 5 d (≥ 2 years)	Preferably in pharmaceutical suspension, drink plenty of fluids

An ear swab is recommended in case of treatment failure.

1.2.2 Otorrhea with tympanostomy tube

- Purely seromucous secretion, no foetor: if necessary, local therapy with **H_2O_2 3% solution**
- In case of persistence Otorrhea, strong foetor:

Ciprofloxacin ED	2 x 4 dr./d (3 mg/ml)	7 (– 10) d	If necessary, in combination with corticosteroids, (e.g., fluocinolone acetonide)

Consider referral to ENT specialist.

1.3 Otitis externa

- Consider referral to ENT specialist: Ointment strips, e.g., **betamethasone/gentamicin** for 1-2 days or alternatively:

Ciprofloxacin ED	2 x 4 dr./d (3 mg/ml)	7 (– 10) d	If necessary, in combination with corticosteroids, (e.g., fluocinolone acetonide)

In case of severe *periauricular swelling* and fever (caution: signs of otitis externa maligna): refer to hospital immediately.

1.4 Acute sinusitis

- Symptomatic therapy (NSAR, decongestants) is usually sufficient, if necessary **mometasone NS** 2 x 2 spray hubs/ nostril/day (5 - 10 d).
- Do not offer AB therapy only on the basis of "purulent" secretion/sputum.
- AB for severe and/or persistent disease (>10 d):

Amoxicillin	50 mg/kgBW/d (max. 3 g) in 2-3 SD	5 (– 10) d	Preferably in pharmaceutical suspension, drink plenty of fluids

1.5 Acute bacterial Lymphadenitis colli

- Usually unilateral, painful on palpation, reddened, warm.
- In case of lack of improvement with oral AB or if abscess formation is suspected: sonography, hospitalization for intravenous therapy and/or surgery (abscess drainage).

**Amoxicillin + Clavulanic acid**	50+12,5 mg/kgBW/d (max. 3,75 g) in 3 SD	7 (– 10) d	Depending upon the clinical course, preferably in suspension, drink plenty of fluids
Cefadroxil	50(-100) mg/kgBW/d (max. 4 g) in 2 SD	7 (– 10) d	Depending upon the clinical course

1.6 Pseudocroup, laryngitis, acute (obstructive) bronchitis, RSV bronchiolitis, influenza

- Do not offer AB therapy as a general rule.

1.7 Community-acquired pneumonia

- If **viral pneumonia** is suspected (preschool age, good general condition, bronchial obstruction), consider laboratory investigation. Do not offer AB therapy, but rather closely monitor **and follow-up**, **anti-obstructive therapy** if necessary.
- If **bacterial pneumonia** is suspected > 6 Mo, (if < 6 Mo, hospitalization and parenteral therapy recommended!):

Amoxicillin	50 mg/kgBW/d (max. 3 g) in 2-3 SD	(3–) 5 d	Preferably in pharmaceutical suspension, drink plenty of fluids

- No indication for chest X-ray in uncomplicated cases
- If **mycoplasma pneumonia** is suspected and there are imminent signs of illness: age > 5 years, epidemiology (incubation period 1-3 weeks), dry cough, obstruction, protracted course. Consider mycoplasma PCR in unclear situations.

Clarithromycin	15 mg/kgBW/d (max. 1 g) in 2 SD	7 (- 10) d	≤ 7 years old
**Doxycycline**	1st day: 4 mg/kgBW/d (max. 200 mg); from day 2 onward: 2 mg/kgBW/d (max. 100 mg) in 1 SD	7 (- 10) d	> 8 years old; do not take with dairy products, avoid sun exposure

1.8 Whooping cough (Pertussis)

- AB ends infectivity / transmission of infection within 5 days, but only shortens the course of the disease when treatment is started by the time of the early convulsive stage.
- Indication: within 3 weeks of the onset of cough or if positive PCR test. Start preventive antibiotics (prophylaxis), especially in the environment of incompletely vaccinated infants or children with underlying cardiac or pulmonary diseases.

**Clarithromycin**	15 mg/kgBW/d (max. 1 g) in 2 SD	7 d	> 2nd MoA

- **Pertussis vaccination (TdaP)** during pregnancy (2nd to 3rd trimester) and in adults (especially parents).

2. Urinary tract infections

2.1 Uncomplicated cystitis

- If the diagnosis is unclear (e.g., DD vulvitis), in case of mild symptoms without fever, no AB therapy and **drinking plenty of fluids,** as well as **short-term follow-up**; **urine culture** recommended.

**Trimethoprim**	6 mg/kgBW/d (max. 400 mg) in 2 SD	3 – 5 d	Take local AB resistance into account
Nitrofurantoin	5 mg/kgBW/d (max. 200 mg) in 2 SD	3 – 5 d	Not suitable in pyelonephritis
Fosfomycine	1 x 3 g (2 hours after dinner, after urination, delay micturition, limit fluids)	1 d	Uncomplicated cystitis in girls ≥ 12 years and > 50 kgKG

- Alternative AB for resistant pathogens when ≥ 6 yrs old: Pivmecillinam 20 - 40 mg/kgBW/d in 3 SD p.o. (max. 1.2 g) 3 – 5 d or Nitroxoline (10 – 20 mg/kgBW/d in 3 SD p.o.; approved from 3 y, available from 14 y).

2.2 Pyelonephritis

- **Uncomplicated pyelonephritis from 4-6 MoA: urine culture!**
- If possible, de-escalation of therapy after urine culture result to e.g., trimethoprim, amoxicillin or cefaclor

**Cefixime**	10 mg/kgBW/d (max. 400 mg) in 1-2 SD	7 (– 10) d	
Cefpodoxime	10 mg/kgBW/d (max. 400 mg) in 2 SD	7 (– 10) d	

- Alternative AB for resistant pathogens ≥ 6 years old, off-label (approval only for cystitis): Pivmecillinam 30 - 40 mg/kgBW/d in 3 SD (TID) p.o. (max. 1.2 g) 7 (-10) d
- **Complicated** pyelonephritis (< 4-6 MoA, pathological urinary tract, poor clinical condition): **hospitalization, parenteral therapy**

2.3 Prophylaxis for urinary tract infections

- Strict indication

**Trimethoprim**	2 mg/kgBW/d (max. 100 mg) in 1 SD	max. 6 Mo	> 6 WoA
Nitrofurantoin	1 mg/kgBW/d (max. 50 mg) in 1 SD	max. 6 Mo	> 3 MoA approved in the absence of an alternative AB
Cefaclor	10 mg/kgBW/d (max. 0,5 g) in 1 SD		< 6 WoA

- Avoid cephalosporins if possible due to the development of resistance (e.g., ESBL-E. coli)!

3. Skin infections

Antiseptic (and **anti-inflammatory**) **local therapy** when possible: **dexpanthenol + chlorhexidin**, **octenidine 0.1%**, **chlorhexidine gluconate cream 0.5%/1%**, **polihexanide solution/gel 0.04%**, **clioquinol cream** (limited area and duration for infants)

3.1 Superinfected atopic eczema

- **Antiseptic** (see above) and **anti-inflammatory local therapy**: e.g., **prednicarbate cream** with **octenidine 0.1%** additive NRF 11.145 (< 6 MoA and face **0.08%,** < 12 MoA **0.15%,** ≥ 12 MoA **0.25%**)
- Consider AB therapy when >10% of body surface is affected, fever, poor clinical condition, failure to improve on local therapy: see impetigo contagiosa

3.2 Impetigo contagiosa

**Antiseptic**	+ consistent hygiene precautions	By course	Sufficient when findings are limited
**Cefadroxil**	50 mg/kgBW/d (max. 2 g) in 2 SD	5 (– 7) d	For extensive findings; alternative: Cefaclor
Clindamycin	30 mg/kgBW/d (max. 1,8 g) in 3 SD	5 (– 7) d	For extensive findings
Cotrimoxazole	30 mg/kgBW/d (max. 1,92 g) in 2 SD	5 (– 7) d	For extensive findings

3.3 “Infected" insect bites

- In case of *pronounced reaction* and "incipient" lymphangitis: compresses (gauze pad) with **antiseptics, immobilization, follow-up**
- If **lymphangitis is certain**: systemic AB therapy for 3 - 5 days (see impetigo contagiosa)

3.4 Perianal GAS dermatitis

- Rapid **Strep A test** or **culture** if necessary: moderate sensitivity, high specificity

Penicillin V	100.000 IU/kgBW/d (max. 3 Mio. IU) in 2-3 SD	10 d	Do not take with meals
Benzathin-Penicillin	50.000 E/kgBW/d (max. 1,5 Mio. E) in 2 SD	10 d	
Cefadroxil	50 mg/kgBW/d (max. 2 g) in 2 SD	7 d	alternative: Cefaclor

3.5 Lyme disease

- **Erythema migrans (EM):** Clinically variable skin rash that begins (3)-7-14-(30) days after tick bite; the rash begins at the site of the bite and expands centrifugally and gradually: erythema (at least 5 cm) with prominent edges (bulls eye)
- Serology may only be useful for multiple EM, not suitable for monitoring of therapy
- Duration of therapy: single EM 10(-14) d, multiple EM or general symptoms 14 - 21 d (depending on duration and severity)
- **Lymphocytoma: serology** recommended/necessary, duration of therapy 14 - 21 days (depending on duration and severity)

Amoxicillin	50 mg/kgBW/d (max. 3 g) in 3 SD	See above	Preferably in pharmaceutical suspension, drink plenty of fluids
**Doxycycline**	4 mg/kgBW/d (max. 200 mg) in 1 SD	See above	Only for ≥ 8 years old, do not take with dairy products, avoid sun exposure

A tick remnant ("head") is not infectious and does not need to be removed; disinfect the wound. No indication for examination of a removed tick for pathogens.

4. Ophthalmic infections

- Strict indication for antibiotic eye drops, e.g., due to indirect effect on nasopharyngeal flora.

4.1 Lacrimal duct stenosis

- No AB therapy recommended.

4.2 Purulent conjunctivitis

- Self-limiting disease, symptomatic therapy is usually sufficient, daycare attendance possible; **AB therapy only indicated if symptomatic therapy fails.**

**Symptomatic therapy**: Clean eyes with water regularly			
Gentamicin OD	1 dr per eye, 4 x /d	until symptoms improve (max. 4 - 5 d)	≥ 2 MoA

Consider chlamydia and gonococci in case of conjunctivitis during the 1st MoA

4.3 Hordeolum

- If necessary, **moist heat**, possibly **bibrocathol 2%** OO 3-5 x daily, no AB therapy.

5. Intestinal infections

5.1 (Hemorrhagic) gastroenteritis

- No AB therapy is usually recommended. Only indicated if **shigella, Salmonella typhi/paratyphi, V. cholerae** and **G. lamblia** are detected, as well as in **septicemia, underlying diseases or immunosuppression**.

6. Surgical infections

6.1 Panaritium, abscess, superficial wounds, minor wound infections, balanitis

- Surgical and antiseptic therapy, no local or systemic AB therapy is usually recommended.

6.2 Animal or human bite wounds

- Surgical and antiseptic therapy, superficial bite wounds: no AB therapy, just observation. Otherwise:

Amoxicillin + Clavulanic acid	50+12,5 mg/kgBW/d (max. 3,75 g) in 3 SD	5 – 10 d	Check **tetanus vaccination status!** Preferably in pharmaceutical suspension, drink plenty of fluids

Rabies vaccination: Terrestrial Germany is currently rabies-free; strict indications: e.g., bat bite, illegally imported animal, bite in a rabies-contaminated foreign country.

Abbreviations

**AB** = antibiotics	**ED** = ear drops	**kgBW** = kilogramm body weight	**OD** = ophthalmic drops
**D** = days	**GAS** = group A streptococcus	**MoA** = months of age	**OO** = ophthalmic ointment
**DD** = differential diagnosis	**Ind** = indication	**Mo** = months	**SD** = single dose
**dr** = drops	**IU** = international units	**NS** = nasal spray	**WoA** = weeks of age

Publisher: Working group "Antibiotic Stewardship (ABS) in Outpatient Pediatrics" (ABSaP):
- Antibiotic therapy in Bielefeld (AnTiB, Ärztenetz Bielefeld)
- Professional Association of Pediatricians and Adolescent Physicians (BVKJ)
- German Society for Pediatric Infectious Diseases (DGPI)
These recommendations represent a revision of "Antibiotic Therapy in Bielefeld - AnTiB Paed 2018"
Content available under the license CC BY-NC-SA 3.0 DE (https://creativecommons.org/licenses/by-nc-sa/3.0/de/)
Further information: www.antib.de, www.dgpi.de
Contact: abs-ambulante-paediatrie@dgpi.de **Version**: March 2024
